# Impacts of dispersants on microbial communities and ecological systems

**DOI:** 10.1007/s00253-022-12332-z

**Published:** 2023-01-17

**Authors:** Stephen M. Techtmann, Jorge Santo Domingo, Robyn Conmy, Mace Barron

**Affiliations:** 1 Department of Biological Sciences, Michigan Technological University, Houghton, MI, USA; 2 Office of Research and Development, U.S. Environmental Protection Agency, Cincinnati, OH, USA; 3 Office of Research and Development, U.S. Environmental Protection Agency, Gulf Breeze, FL, USA

**Keywords:** Oil biodegradation, Dispersants, Bioremediation, Toxicity, Microbial community

## Abstract

Accidental oil spills can result in catastrophic ecological insults and therefore require rapid intervention to mitigate the potential impacts to aquatic ecosystems. One of the largest oil spills, known as the Deepwater Horizon oil spill, occurred in the Spring of 2010 near the coast of Louisiana (USA) due to an explosion during oil drilling activities. Millions of gallons of oil were released into the Gulf of Mexico, impacting thousands of ocean miles and coastal areas linked to the gulf. Among the actions taken during the remediation efforts was the unprecedented large use of Corexit dispersants, including at the subsurface to prevent oil from reaching the surface. While there is evidence that dispersants can accelerate the biodegradation of oil, reports on their potential toxicity to aquatic biota and to microbial functions have also been documented. In this review, we will examine the most recent literature on the impact of dispersants on microbial communities implicated in oil degradation and overall ecological networks. The primary focus will be on studies using Corexit but other dispersants will be discussed if data are available. We will share the literature gaps identified and discuss future work that is needed to reconcile some of the discrepancies found on the effectiveness of dispersants on oil degradation and their potential toxicity.

## Introduction

Accidental releases of oil have had dramatic impacts on aquatic ecosystems in both marine and freshwater environments (Atlas and Hazen 2011; Hazen et al. 2016). Oil spills need immediate mitigation considering the impacts to exposed biota. Several remediation approaches have been utilized over the years to decrease their impacts. In general, they can take the form of physical, chemical, or biological approaches (Fingas 2016; Techtmann and Hazen 2016). There are strengths and limitations to each of these approaches. Here, we review the recent literature related to the impact of chemical dispersants on the microorganisms involved in oil biodegradation.

Chemical dispersants are formulations that contain surfactants as the active ingredient. Surfactants reduce the oil–water interfacial tension, facilitating the formation of small oil droplets (< 100um) that can be entrained into the water column while minimizing recoalescence (Li and Garrett 1998; Prince 2015, 2023). During the *Deepwater Horizon* (DWH) oil spill, 2.1 million gallons of chemical dispersants were applied to both floating surface slicks and submerged oil released from the damaged well head pipe in an attempt to break up formed slicks and also prevent the released oil from accumulating at the surface (King et al. 2015; Lubchenco et al. 2012). This subsea addition of dispersant was hypothesized to disperse the Louisiana light sweet crude oil through the water column and limit the amount of oil that reached the surface to form a slick (Barron 2012).

The use of chemical dispersants as a response strategy is intimately linked with microbially mediated biodegradation of oil. Once the oil is dispersed into the water column, biodegradation is relied upon to remove the more labile fractions of the oil from the system provided that sufficient nutrients are available. Due to the poor solubility of chemicals in oil and their hydrophobicity, oil biodegradation rates are often limited by the ability of microorganisms to access the oil. Therefore, it is hypothesized that chemical dispersants can increase oil biodegradation through the increase of the oil surface-to-volume ratio (Brakstad et al. 2015; Venosa and Holder 2007). With increased surface area, there are more sites for colonization by oil-degrading microorganisms. There have been varying reports on the degree to which dispersants stimulate oil biodegradation. Many studies have reported a positive or neutral effect of dispersants on oil biodegradation (Brakstad et al. 2018a; Sun et al. 2019; Techtmann et al. 2017; Tremblay et al. 2017), whereas others have reported a negative impact of dispersant addition on oil biodegradation (Kleindienst et al. 2015b; Rughöft et al. 2020). However, the volume of oil, dispersant dosing, consortia/cultures, and experimental conditions vary among these studies. Here, we seek to review the recent literature related to the impact of chemical dispersants on both oil biodegradation and microbial community structure. The primary focus will be on the chemical dispersant Corexit 9500 due to its large application during the DWH oil spill and greater number of research studies conducted in the laboratory compared to other dispersants. However, there are other formulations of chemical dispersants that have been explored, some of which compare the biodegradation rates of oil dosed with multiple dispersant products (Prince et al. 2016; Zhang 2016; Zolfaghari-Baghbaderani et al. 2012). It should also be noted that in recent years, Corexit dispersant products are predominantly stockpiled in the USA, although there are currently 19 dispersants listed for use in US waters in the National Oil and Hazardous Substances Pollution Contingency Plan Product Schedule (NCPPS) technical notebook (40 CFR § 300.905, 1994). The goal of this review is to summarize the current knowledge related to the impact of dispersants on oil biodegradation with a focus on recent studies on the environmental impacts of dispersant application, comparison of the impacts of dispersants on oil biodegradation in the Gulf of Mexico with other oceanic settings, and summary of recent work on impacts of non-Corexit dispersant formulation on oil biodegradation.

## Introduction to oil biodegradation

Over 175 genera have been identified with the ability to metabolize the various components of crude oil (Hazen et al. 2016). These genera are associated with diverse microbial taxa. In the oceans, members of the class Proteobacteria, especially Alpha- and Gammaproteobacteria, are often the dominant taxa that respond to oil spills (Hazen et al. 2016; Hazen and Techtmann 2018; King et al. 2015). Many of the dominant oil degraders that responded to the DWH oil spill were genera such as *Alcanivorax*, *Marinobacter*, *Cycloclasticus*, *Colwellia*, *Alteromonas*, and members of the order *Oceanospirilales*, altogether comprising up to 80–90% of the recovered 16S rRNA gene sequences from the bacterial fraction in the deep water oil plume during the DWH oil spill (Mason et al. 2012). In addition to these Gammaproteobacteria, Alphaproteobacteria have been shown to respond to released oil in some marine settings, especially in surface water (King et al. 2015; Sun et al. 2019). Many of these taxa have been relied upon to break down released oil in response to oil spills as part of the natural attenuation of the released oil.

**Fig. 1:**
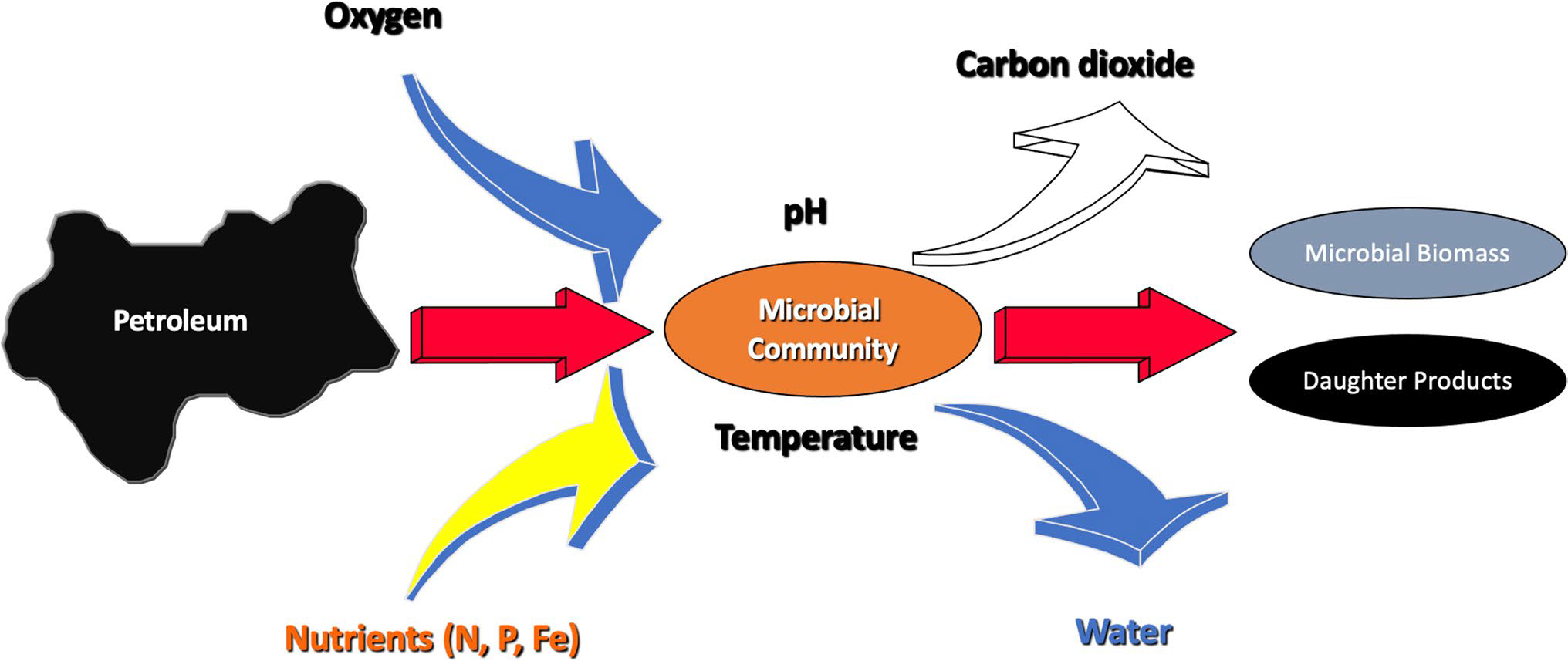
Conceptual model of biodegradation and the various factors that affect the rates of oil biodegradation. Adapted from (Hazen et al. 2016)

Aerobic oil biodegradation can be strongly impacted by the availability of oxygen as well as other nutrients such as nitrogen and phosphorous (Fig. 1). Temperature is also believed to be a major constraint on oil biodegradation as the rates many biological reactions are greatly decreased at low temperatures (Zobell 1934). However, evidence from the deep water in the DWH spill as well as recent work in the Arctic have shown that oil can be metabolized rapidly under very cold conditions, suggesting that there are cold-adapted microorganisms that are capable of rapidly breaking down oil (Campo et al. 2013; McFarlin et al. 2014, 2018; Prince et al. 2013). These cold-adapted bacteria are believed to be the first responders in cold environments such as the deep ocean and polar settings. In addition to environmental conditions impacting the rates of oil biodegradation, physical phenomena such as water solubility and dissolution might also act to slow biodegradation.

Due to the limited solubility of many components of crude oil, oil biodegradation often is initiated by colonization of the oil–water interface by oil-degrading bacteria (Macnaughton et al. 2003). The process of colonization may be a limiting constraint during an oil spill as the oil coalesces to form a slick. To overcome this rate-limiting step, many oil-utilizing bacteria produce biosurfactants, which can help to solubilize oil for increased colonization. In a similar fashion, chemical dispersant application is believed to stimulate biodegradation through increasing the surface area of the oil and provide more access to the oil–water interface for oil-degrading microbes to colonize.

## Ecological effects of dispersants

Dispersants contain mixtures of solvents (in some cases light petroleum distillates) and surfactants. For examples, Corexit EC9500 and to a lesser extent Corexit EC9527 (Nalco Holding Company, Inc.; Naperville, IL) were applied during the DWH oil spill (King et al. 2015). Both dispersants typically contain approximately 18% of the surfactant Dioctyl sulfosuccinate (DOSS), 27% of the non-ionic surfactants tween 80 and tween 85, and approximately 55% carrier solvents such as ethylene and dipropylene glycol as well as petroleum distillates (McFarlin et al. 2018; Staff et al. 2005). While the solvents appear to be readily biodegradable, there are varying reports of the biodegradability and persistence of the main surfactant in Corexit 9500 (i.e., DOSS). Some studies have reported the biodegradability of DOSS (Brakstad et al. 2018b; Campo et al. 2013), whereas other studies reported that DOSS persisted in the Gulf of Mexico deep water at detectable levels long-term post release (Gray et al. 2014; White et al. 2014). This is in part due to slow metabolic rates in cold temperatures and/or high hydrostatic pressures in the deep sea, and thus may be used as an indicator of persistence of the dispersants in some natural waters. However, DOSS from the DWH spill was not found to persist in surface waters or nearshore environments, where concentrations of oil and DOSS in tar balls and patties were patchy and inconsistent (White et al. 2014) and chemical fingerprinting of waters pointed to stormwater pollution as the source of DOSS (Hayworth and Clement 2012). The reported long-term persistence of components of Corexit in the Gulf of Mexico implies that while the use of dispersants can be viewed as a practical near-term approach in spill response efforts, the long-term consequences of dispersant use need to be better understood (Lee et al. 2016).

Ecological concerns with dispersant use include increased bioavailability of oil and hazards to a wide array of organisms. Dispersant toxicity has been shown for crustaceans, fish, corals, anemones, mollusks, amphipods, polychaetes, algae, and ciliates to mention a few nonmicrobial taxa (Almeda et al. 2014b; Barron et al. 2013; DeLorenzo et al. 2017; Durier et al. 2021; Shafir et al. 2007; Toyota et al. 2017). In general, these studies have shown various degrees of toxicity to different taxa of aquatic organisms, with some species showing high sensitivity. For example, some microzooplankton species have been reported to be among the most sensitive species to Corexit 9500 (Almeda et al. 2014a). As they serve as important food sources of fish, the impact of dispersed oil droplets on microzooplankton has further implications in natural food webs (Almeda et al. 2014a).

Most of the recent research on dispersed oil toxicity has been focused on Corexit 9500A due in part to its large application in response to the DWH oil spill. However, other dispersants have been evaluated and shown different levels of ecological hazard (Shafir et al. 2007). A study after the DWH oil spill showed that Corexit 9500A had generally similar toxicity as the seven other tested dispersants, and that dispersants alone were less toxic than the oil/dispersant mixtures (Hemmer et al. 2011).

While it is known that dispersants themselves can be toxic, the effect of adding dispersants to petroleum has been reported to either increase or have no effect on the toxicity of dispersed oil to aquatic organisms. The current general scientific consensus is that dispersants do not change the intrinsic acute toxicity of oil (Lee et al. 2016; NASEM (2020). A meta-analysis performed as part of NASEM (2020) suggested that generally there was no difference in the toxicity of oil alone and chemically dispersed oils at dispersant concentrations below 100 ppm. Under field conditions, dispersant concentrations are expected to generally be below 1 ppm and not exceed toxicity thresholds for dispersant alone (e.g., Bejarano 2018). However, the results of toxicity studies can be confounded by the fact that in many cases methods for controlling and measuring concentrations of crude oil in exposure tests are difficult or inadequately reported. Also, different methods, endpoints, and test durations are used to determine dispersant and oil toxicity and therefore studies can generate different conclusions. Moreover, some species are more sensitive than others, suggesting that such generalizations need to be taken with some precaution. Standardizing toxicity testing for oil and spill response agents have been recently recommended to improve comparability across studies (Hodson et al. 2019). Species sensitivity distributions, which are probability distributions of toxicity values for species assemblages, provide a tool for estimating toxicity to aquatic species when species or life stage–specific sensitivity is not available (Barron et al. 2013; Bejarano 2018).

Little is known about the effect of dispersants on sublethal or chronic toxicity of oil, and the impacts of chemically dispersed versus only physically dispersed oil on ecological systems are largely unknown. Dispersants increase the bioavailability of oil in the water column, potentially increasing the exposure of aquatic organisms to petroleum hydrocarbons. Dispersants can also increase the proportion of three ring and larger PAHs compared to oil alone, potentially increasing sublethal or chronic toxicity. Whereas napthalenes and other small PAHs have acute toxicity at part per million concentrations, larger PAHs may cause developmental and chronic toxicity at concentrations in the low part per billions (Barron et al. 2004). Differences in species sensitivity and oil exposure may have important effects at different trophic levels that can lead to impacts at the ecosystem level (Barron et al. 2020). For example, dispersant/ oil mixtures can impact ciliates that act as normal predators of dinoflagellates potentially resulting in the formation of harmful algal blooms (Almeda et al. 2014a). Dispersant-treated crude oil was reported to reduce egg production rates and egg hatching of different copepods which are zooplankton important in marine food webs (Almeda et al. 2014a). In studies evaluating third generation dispersants (Inipol IP-90, Petrotech PTI-25, Bioreico R-93, Biosolve and Emulgal C-100), it was noted that they cause further decrease in settlement rates of coral larvae than oil water-soluble fractions (Epstein et al. 2000). Results on the effect of coral larvae settlement for additional non-Corexit dispersants were recently reported by Negri et al. (2018). However, in general, less is known for dispersants other than Corexit as far as persistence and potential ecosystems effects. The limited available information and inferences from acute toxicity studies suggests that dispersants may increase the chronic toxicity of oil because of changes in bioavailability and exposure to larger PAHs, but additional research is needed to better understand the magnitude and scale of any effects.

With recent advances in high throughput sequencing, researchers have looked at transcriptional profiles of a variety of aquatic biota to study potential toxic effects of dispersant and oils amended with dispersants. Such approach provides another level of assessment, particularly in cases when chemical exposure does not induce acute mortality or phenotypic malformations. As with conventional methods, many gene expression studies have shown that dispersants have lesser or no effect when compared to oil-amended exposures. However, different patterns have been observed. For example, Hook and Osborn (Hook and Osborn 2012) showed very similar gene expression profiles in diatoms regardless of treatment, although based on observed membrane damage, they were more sensitive to dispersants and dispersed oil than to oil-only treatments. In contrast, two coral species had the greatest difference in the number of expressed genes in mesocosms containing dispersant (Corexit 9500) than those with oil only (DeLeo et al. 2021). In another study, Li et al. (2021) showed that zebrafish embryo-larvae had the highest number of differentially expressed genes when treated with sublethal concentrations of Chemically-Enhanced Water Accommodated Fraction (CEWAF) for 120 h, while the lowest when exposed to the dispersant GM-2. Overall, studying gene expression can lead to specific pathways that are impacted by the chemical of interest and identify genes that may be used for screening purposes.

Toxicity of dispersant towards microorganisms has been studied to a lesser extent and in many cases the evidence is presented in the form of relative abundance of some populations within complex microbial communities. In other cases, studies have been conducted with pure cultures to target specific functions. For example, studies using environmentally relevant levels of Corexit 9500A indicated no toxic effect on the nitrifying species *Nitrosomonas europaea* (Radniecki et al. 2013). However, oil-amended samples revealed increased toxicity based on chemical oxygen demands levels. The impact of dispersant applications on nitrification inhibition in natural systems could lead to increases in ammonia availability, in turn stimulating the growth of many heterotrophs, including hydrocarbon degraders (Urakawa et al. 2019).

Using pure cultures of two oil-degrading bacteria, Rahsepar et al. (2017) showed that Corexit can reduce oil biodegradation in the presence of alginate particles and kaolin clay by increasing the solubility of toxic hydrocarbons (e.g., BTEX). The authors suggested that a component of Corexit (DOSS) may interact with the alginate particles that were used to simulate marine snow slowing down bacterial adherence and thus the formation of oil-degrading bacterial biofilms. Additional work has shown that Corexit impacts bacterial isolates in a species-specific manner. Overholt et al. (2016) demonstrated that growth and oil biodegradation potential of *Acinetobacter* were inhibited by addition of Corexit, whereas growth of *Alcanivorax* was stimulated in the presence of Corexit.

While studies using seawater amended mesocosms have shown that some of the components present in Corexit degrade within days in seawater (Gofstein et al. 2020) and that Corexit can increase the number of hydrocarbon-degrading genes (McFarlin et al. 2018), delay in DOSS degradation in the presence of oil has also been reported (Gofstein et al. 2020). These results highlight the complex nature of dispersant, microbial, and colloidal interactions when dispersants are used to enhance oil degradation.

Other pure culture studies have shown the potential impact of Corexit on hydrocarbon degraders. For example, Rughoft et al. (2021) showed that Corexit impacted hydrocarbon metabolism, chemotactic motility, and biofilm formation of *Marinobacter* sp TT1. *Marinobacter* species are known for their capability of degrading hydrocarbons. The same research group showed that Corexit could also impact the growth of this strain when previously grown under preadapted low concentrations of n-hexadecane (i.e., simulating starving conditions) vs non-starved cells (Rughöft et al. 2020). These studies suggest that dispersants may have detrimental impacts to some oil-degrading bacteria under natural environmental conditions. However, several studies have shown that other oil degraders increased in relative abundance after Corexit was applied suggesting the presence of bacterial taxa in marine waters that are relatively tolerant to Corexit.

## Impact of Corexit 9500 on microbially mediated oil biodegradation in the Gulf of Mexico

There are varying reports on the efficacy of dispersants on oil biodegradation in marine systems. Previous reports have suggested a positive, neutral, or negative impact of Corexit on oil biodegradation (Table 1). For example, a study performed early-on in response to the DWH spill demonstrated that a higher amount of oil was degraded in Corexit-amended microcosms (Bælum et al. 2012). Additional reports using an enriched consortium of oil-degrading bacteria indicated a stimulation of oil biodegradation in Corexit-amended conditions (Campo et al. 2013; Techtmann et al. 2017). These studies showed that the addition of Corexit to microcosms resulted in both increased biodegradation as well as alterations in the microbial community composition. Specifically, in Techtmann et al. (2017), the addition of Corexit resulted in a decrease of *Marinobacter* spp. in the active fraction of the community (based on 16S rRNA gene transcripts) relative to the oil-only condition in microcosms incubated at 25 °C. Alternatively, at 5 °C, there was little change in the microbial community upon addition of Corexit. Furthermore, in samples with Corexit-only, there were elevated levels of *Idiomainacea* in the 25 °C incubations, and elevated levels of *Colwellia* spp. in the 5 °C enrichment. These finding suggest that there may be some members of the community able to consume some of the components of Corexit.

Other work has suggested that the mixing conditions can play a major role in regulating the efficacy of dispersant applications. Sun et al. (2019) performed a lab experiment to determine the impact of mixing conditions on oil biodegradation with and without dispersant. In this study, low mixing conditions were simulated using booms whereas high mixing conditions were mixed at 100 rpm with no booms. Sun et al. (2019) demonstrated that under low mixing conditions, the addition of dispersant did not affect oil biodegradation rates relative to the no-dispersant control. However, under high mixing conditions, there was a substantial increase enhancement of oil biodegradation decreasing the half-life of petroleum hydrocarbons from 15.4 to 8.8 days. Furthermore, the addition of Corexit dramatically altered not only the metabolically active microbial community composition, but also the composition of the nitrogen-fixing bacterial community.

In contrast to studies that have shown enhanced biodegradation upon Corexit addition, other studies have reported no enhancement or even a negative effect of Corexit on oil biodegradation (Kleindienst et al. 2015b). For example, Kleindienst and colleagues demonstrated that Corexit addition had a negative impact on both cell growth and oil biodegradation relative to treatments with only the water accommodated fraction (WAF) of oil. In Kleindienst et al. (2015b), radiotracers were used to determine rates of hydrocarbon degradation. They found that hexadecane degradation was higher in the WAF treatment compared to the chemically enhanced WAF (CEWAF) treatment with oil and Corexit 9500. The authors concluded that dispersants suppressed hexadecane degradation. These contrasting results have the potential to complicate a proper understanding of the impact of Corexit on oil biodegradation.

When seeking to reconcile these disparate data, it is important to consider experimental design and methods for assessing the role of dispersants in oil biodegradation. Much of the work that shows a positive effect of Corexit on oil biodegradation involves the use of microcosms where oil is directly added to water or culture medium/sea water and the formation of a slick occurs. Therefore, the addition of Corexit results in dispersion of the oil slick into the water. As colonization is a key step in oil biodegradation, Corexit may enhance the rate of oil biodegradation due to the ability to increase the surface-to-volume ratio of the oil from the slick. The studies that have reported a negative impact of Corexit often use the WAF of oil. WAF is routinely used in toxicity studies to determine the impact of water-soluble constituents of oil on biological communities. The use of the WAF minimizes the importance of the activity of dispersants in breaking up of the slick and therefore, the positive enhancement of dispersants on oil biodegradation is minimized. These differences in experimental design have the potential to impact the outcome of the studies and may explain the differences in the observed impact of dispersant on oil biodegradation. Clearly, one important future research goal is to discuss and clarify a consistent experimental approach for laboratory settings that could be applied and allow for comparative studies of the impact of dispersants on oil biodegradation. Furthermore, it is important to employ methods for simulating realistic conditions in the laboratory that would be analogous to the conditions observed in the environment.

Despite the differences in trends related to the impact of Corexit on oil biodegradation, there is consistency in that Corexit addition results in altered microbial community composition. For example, a few studies reported that the addition of Corexit resulted in a distinct population of bacteria, with members of the *Colwellia* being substantially altered by the presence of Corexit (Kleindienst et al. 2015a, b). It has also been demonstrated that the addition of Corexit results in a strong stimulation of several microbial groups including *Colwellia* under cold conditions (Techtmann et al. 2017). In the latter study, Techtmann et al. treated a defined consortium of oil-degrading microbes with Corexit alone, oil, and oil plus Corexit and generated 16S rRNA gene sequencing libraries using both the DNA and RNA extracts to determine the impact on the total community. The sequencing data showed that the addition of Corexit alone resulted in a strong stimulation of *Colwellia* spp. Furthermore, it was shown that there appeared to be a strain-specificity in relation to the impact of Corexit (based on Operation Taxonomic Units (OTU) clustered at 97% sequence identity). Specifically, some OTUs classified as *Alcanivorax* were stimulated in response to Corexit addition and potentially could be contributing to increased biodegradation or stimulated by the hydrocarbon components of Corexit. Other *Alcanivorax* OTUs decreased in relative abundance after the presence of Corexit. Similar findings were observed by others with samples from the Gulf of Mexico (Overholt et al. 2016).

There are data suggesting that the stimulation of microbes upon Corexit addition is in part due to the ability of microbes to degrade Corexit. For example, some *Colwellia* isolates can breakdown many of the components of Corexit, suggesting their ability to use Corexit as a carbon source (Chakraborty et al. 2012). Corexit contains a diverse mixture of carbon sources including Tween compounds, polyethylene glycol, as well as the surfactant DOSS. Bacteria have been shown to use many of these components as carbon sources and there are several reports of biodegradation of Corexit components (Campo et al. 2013). Furthermore, recent studies have found that Corexit addition stimulates that growth of known hydrocarbon-degrading bacteria. This stimulation has led to the hypothesis that Corexit may stimulate biodegradation through a priming-like effect (Schreiber et al. 2019). As some components of Corexit include relatively easy to break down hydrocarbons, it encourages the growth of oil-degrading microbes increasing their abundance and allowing for faster oil biodegradation rates.

Despite the number of findings demonstrating an impact of Corexit on the microbial community, these studies have all been performed using a microcosm- or mesocosm-based approach. The use of microcosms is a very common tool for simulating the effects of oil spills on microbial populations. However, some artifacts can be introduced during microcosm experiments such as bottleneck effects by performing the experiments in closed systems, or founder effects as the initial populations can strongly impact the microbial composition in the end of these incubations. All of these factors confound the inference of the impact of Corexit on natural microbial communities.

## Impact of Corexit 9500 on microbially mediated oil biodegradation in other oceanic regions

Many studies of the impact of dispersants on oil-degrading microbial communities were performed in response to the DWH spill and have focused on the samples from the Gulf of Mexico. However, it is important to consider differences in the microbial response to dispersants in diverse oceanic contexts as dispersant applications are considered as a potential spill response strategy in systems outside of the Gulf of Mexico. Environmental conditions and microbial community composition can vary greatly from one location to another or even at the same site as a function of depth (Hazen and Techtmann 2018; Miller et al. 2020). Many hydrocarbon basins can differ greatly in salinity with basins such as the Eastern Mediterranean having salinities near 39 psu and the Caspian Sea has much lower salinities near 11 PSU in the Southern Caspian (Miller et al. 2019; Techtmann et al. 2015). Salinity is known to impact the effectiveness of dispersants (Cao et al. 2022; Chandrasekar et al. 2006; Clayton et al. 2020; Fingas 1991). Previous studies have shown that dispersant activity increased with increasing salinity. However, other factors such as oil type, weathering of the crude oil, and temperature can all play a role in impacting the effectiveness of dispersants (Chandrasekar et al. 2006). Furthermore, pressure has been shown to affect oil biodegradation with higher pressures often decreasing the extent of oil biodegradation (Marietou et al. 2018; Nguyen et al. 2018). These findings indicate the importance of exploring the impact of dispersants on biodegradation and microbial communities in basins outside of the Gulf of Mexico.

The impact of Corexit has been explored in locations such as the Arctic, Norwegian sea water, and coastal Canadian water, among others. Several studies on the role of oil droplet size on oil biodegradation were conducted in samples collected for Norwegian sea water. Studies that have reported a positive effect of dispersants often attribute this to the ability of dispersants to decrease the droplet size of the oil, thus increasing the surface area available for colonization. This hypothesis was tested and results showed oil droplets of a size of 10 μm degraded at a faster rate compared to that of 30 μm (Brakstad et al. 2015). While these studies demonstrate the importance of droplet size on oil biodegradation, as droplet size decrease a point is reached where the degradation rates would become slower due to a decrease in encounter rate (Juarez et al. 2016).

Additional studies have explored the overall impact of Corexit 9500 on the microbial response to oil and the oil biodegradation potential of microbial communities from Norwegian sea water (Ribicic et al. 2018). These studies found that chemically dispersed crude oil is rapidly biodegraded by bacteria from Arctic and Norwegian Fjord sea water. The microbial community in these samples showed that relatives of the oil-degrading genus *Oleispira* were abundant at the beginning of the experiment in water collected from the Arctic and increased in abundance in both Arctic and Norwegian Fjord samples amended with chemically dispersed oil.

Other studies have investigated the effect of Corexit on oil biodegradation and Corexit biodegradation in samples from the Arctic. Work by McFarlin et al. (2014) demonstrated that indigenous microbial communities in Arctic seawater are capable of degrading oil in the presence and absence of Corexit at subzero temperatures (McFarlin et al. 2014). They found that between 46 and 61% of the oil was lost during incubations at − 1 °C and 14% of the Corexit 9500 components were mineralized during the 60-day incubation. These results demonstrated that initially there was an enhancement in oil biodegradation in the presence of Corexit, but the difference was minimal with longer incubations. Following on from this work, McFarlin et al. examined the ability of Arctic microbial communities to degrade components of Corexit (McFarlin et al. 2018). They found that microcosms from the Chukchi Sea incubated at 2 °C showed between 33 and 77% degradation of DOSS over the 28-day incubation, while the non-ionic surfactants in Corexit were below detection after 28 days, indicating that even under very cold conditions, the indigenous microbial community in the Arctic is capable of degrading DOSS and other components of Corexit. An additional study examined the interactive effects of Corexit and oil biodegradation (Gofstein et al. 2020). Using a mesocosm-based approach, the rates of oil and Corexit biodegradation by microbial communities from Arctic seawater were examined. Similarly, to the microcosm-based study of McFarlin et al. (2018), the non-ionic surfactant components of Corexit were quickly biodegraded to below the limit of detection after 5 days, whereas DOSS was more persistent. In treatments using dispersed oil, DOSS degradation was slower in the presence of oil than in the Corexit only conditions. Similar sets of bacterial taxa were enriched in the oil, oil and Corexit, and Corexit only conditions leading the authors to conclude that some organisms are capable of consuming both oil and Corexit. Altogether, these findings indicate that the indigenous microbial communities in Arctic seawater can rapidly degrade oil and Corexit compounds even under very cold conditions. These studies suggest that Corexit either shows a positive or negligible effect on oil biodegradation in these experiments.

Recently, many studies have examined the impact of Corexit and other dispersants on the oil biodegradation potential of microbial communities from coastal Canada. One study explored the impact of Corexit EC9500 on oil and gas condensate biodegradation and community composition in samples collected from the east coast of Canada (Tremblay et al. 2017). In this experiment, samples were collected from offshore near oil and gas infrastructure during the summer and winter. Like some studies in the Gulf of Mexico, Tremblay et al. (2017) found that Corexit addition enhanced oil biodegradation. Additionally, they found that Corexit amendments resulted in structural shifts in the microbial community, which is in line with most studies from the Gulf of Mexico. Interestingly, while many studies from deep sea water found that *Colwellia* spp. are enriched in conditions with Corexit, these authors found that abundance and expression of genes from a *Thalassolituus* sp. (a member of the Oceanospirillales) were increased in conditions with Corexit. This study explored the impact of Corexit on gas condensate biodegradation and found that gas condensate is quickly degraded without the need for dispersant addition. These results suggest that the impact of dispersants on oil biodegradation may be different depending on the released hydrocarbons. It also further demonstrates that in most marine environments there is an indigenous population of oil-degrading bacteria that can biodegrade many classes of hydrocarbons.

Further studies were performed examining the impact of Corexit on biodegradation of heavy crude oil (diluted bitumen) (Schreiber et al. 2019). Diluted bitumen is a heavy crude oil derived from oil sand deposits and diluted with gas condensates for ease of transportation. Schreiber et al. (2019) found that while n-alkanes and select PAHs were degraded, most of the PAHs found in diluted bitumen persisted throughout the experiment. Schreiber et al. (2019) also found variability in the response to dispersant with some treatments and conditions showing an enhancement in oil biodegradation in response to Corexit addition and others showing little difference from the oil-only treatments. In particular, the authors found that Corexit addition enhanced degradation of some PAHs in winter microcosms. Because most of the work on Corexit’s impact on oil biodegradation has focused on light crude oils, in response to the Deepwater Horizon oil spill, this work helps to expand our understanding of the impact of Corexit on biodegradation of heavy crude oils.

## Comparison of Corexit with other dispersants

Due to the extensive use of Corexit 9500 during the Deepwater Horizon oil spill, Corexit 9500 is the most well-studied of the chemical dispersants, with the vast majority of the studies focusing on Corexit 9500A. However, there are nineteen distinct products listed on the US National Contingency Plan Product Schedule (www.epa.gov/emergency-response/ncp-product-schedule-products-available-use-oil-spills). All these dispersants have been tested for their toxicity and effectiveness. Toxicity tests include comparison of the toxicity of the dispersant, oil, and oil plus dispersant with two estuarine species: the crustacean *Americamysis bahia* and the fish *Menidia beryllina*. A swirling flask effectiveness test is commonly used to determine the effectiveness of the dispersant at dispersing various types of crude oil. Recent studies have begun to examine alternate dispersants such as Daisic Slickgone NS and Finasol ORS52. Some results have shown that the surfactant components of all of these dispersants are biodegraded in cold (5 °C) seawater by natural microbial communities (Brakstad et al. 2018b). As the concentration of the dispersant was increased, the halflives dramatically increased. The authors concluded that it is essential for studies about the impact of dispersant on biodegradation to be performed at environmentally relevant concentrations. This work demonstrates that surfactants from common dispersants can be biodegraded under cold conditions.

Many oil-degrading bacteria produce biosurfactants as a means of accessing the compounds in oil slicks and droplets. These biosurfactants have been proposed as alternatives to chemical dispersants. A recent study sought to do a cross-comparison between Corexit 9500 and four biosurfactants (surfactins, trehalose lipids, rhamnolipids, and exmulsins) (Cai et al. 2021). They compared the ability of these biosurfactants to disperse Arabian light crude and weather Alaskan North Slope oil. This work found that some of the biosurfactants could perform at similar levels to the commercial chemical dispersant Corexit in terms of dispersion efficiency.

Additional research is needed to fully understand the impact of dispersants other than Corexit on microbial community composition and activity.

## Conclusions and future directions

Dispersants have been used as an important tool in spill response such as during the DWH oil spill and have sparked several studies into their effectiveness and the environmental implications of their use. While dispersants are effective at breaking up slicks, there is great interest into the fate of dispersed oil after the slicks have been broken up. However, recent reports show varying impacts of Corexit on oil biodegradation: many studies have reported a stimulation of oil biodegradation upon dispersant addition in diverse environments while others have reported inhibition. These differences in results could be due to differences in experimental design such as the use of CEWAF versus direct oil addition that have led to contrasting conclusions related to the impact of dispersants on oil biodegradation. Diverse reports regarding the impact of Corexit on microbial community composition are now available and the reality is that the information is limited to few geographic locations. There also is evidence for strain-specific responses to Corexit addition as some members of the same species show a differing response to dispersant addition, but the universality and importance of this observation is not fully understood. There is a need to bring some more consensus to effects of dispersants on oil biodegradation and microbial community composition, a goal that highlights the need for consistency on the experimental design. We believe that this consistency could be achieved through a standardized approach for incorporating biodegradation into dispersant effectiveness tests. These efforts will require improving our understanding of the composition and function of microbial networks responsible for the degradation of different components of dispersants and dispersed-oil fractions. In this regard, the importance of emerging technologies such as next-generation sequencing and high throughput detection of targeted populations via genetic markers, in conjunction with the accelerated improvement of bioinformatics and predictive models, will provide more comprehensive insights on these questions. Furthermore, with most of the work focused on Corexit 9500, there is need to investigate the impact of other dispersants on the NCP product list on oil biodegradation. Overall, dispersants show promise as a tool for oil spill response teams, but there is need for more information to better understand the impact of dispersants on microbial communities and the conditions under which dispersants should be applied. The long-term effects of dispersants and dispersed oil on ecosystem health deserve greater attention in the future.

## Figures and Tables

**Table 1:** Select studies examining the impact of dispersants on microbial community and oil biodegradation

Paper	Inoculum	Dispersant used	Sample collection	Direct oil addition vs. WAF	Overall conclusion

Campo et al. (2013)	Enriched consortium	Corexit 9500	Gulf of Mexico	Direct oil addition	Corexit biodegradation and oil biodegradation occurred at both 25 °C and 5 °C
Techtmann et al. (2017)	Enriched consortium	Corexit 9500	Gulf of Mexico	Direct oil addition	Corexit had a positive effect on oil biodegradation in Corexit amended cultures. Corexit addition affected the active microbial community
Sun et al. (2019)	Seawater	Corexit 9500	Gulf of Mexico (Pensacola beach)	Direct oil addition	Under low mixing conditions little effect of dispersant addition was seen. However, under high mixing conditions, there was an enhancement of biodegradation
Rughoft et al. (2021)	Marinobacter isolate	Corexit 9500	Isolate from the Gulf of Mexico	WAF	Corexit exposure affects hydrocarbon metabolism, chemotactic motility, and biofilm formation of *Marinobacter* isolates
Kleindienst et al. (2015b)	Seawater	Corexit 9500	Gulf of Mexico (Pensacola beach)	WAF	Biodegradation of oil was less in the CEWAF compared to the WAF conditions
Baslum et al. (2012)	Seawater	Corexit 9500	Gulf of Mexico	Direct oil addition	Increased amount of oil was degraded in Corexit amended enrichments
Brakstad et al. (2015)	Seawater	Corexit 9500	Norwegian sea water	Oil droplets of different size	The size of the oil droplets affect biodegradation of oil with smaller droplets having faster oil biodegradation
Ribicic et al. (2018)	Seawater	Corexit 9500	Norwegian sea water	Direct oil addition	These studies found that chemically dispersed crude oil is rapidly biodegraded by bacteria from Arctic and Norwegian Fjord sea water
McFarlin et al. (2014)	Seawater	Corexit 9500	Arctic seawater	Direct oil addition	Initial enhancement of oil biodegradation in the presence of dispersant
Gofstein et al. (2020)	Seawater	Corexit 9500	Arctic seawater	Direct oil addition	Corexit addition did not impact oil biodegradation. Corexit compounds appear to be preferentially degraded over oil
Tremblay et al. (2017)	Seawater	Corexit 9500	Canadian coastal seawater	Direct oil addition	Corexit addition enhanced oil biodegradation
Schreiber et al. (2019)	Seawater	Corexit 9500	Canadian coastal seawater	Direct oil addition	Impact of dispersants was variable between microcosms
Brakstad et al. (2018b)	Seawater	Corexit 9500, Daisic Slickgone NS and Finasol ORS52	Norwegian sea water	Dispersant added to water	Many of the surfactants used in dispersant formulations can be biodegraded in cold seawater by the native microbial community

## Key points

- Chemical dispersants have been applied as a chemical response measure for oil spills.
- The effects of chemical dispersants on microbial communities have been the subject of substantial research.
- This work seeks to review recent work on the impact of chemical dispersants on oil biodegradation, microbial communities, and ecosystems.
